# Multi-expert fusion for state-of-health estimation of lithium-ion batteries

**DOI:** 10.1038/s41598-025-26163-1

**Published:** 2025-11-26

**Authors:** Qinyuan Fan, Guangxing He, Diwang Ruan, Clemens Gühmann

**Affiliations:** 1 Chair of Electronic Measurement and Diagnostic Technology, Technische Universität Berlin, Berlin, 10587 Germany; 2 School of Electronic Engineering and Computer Science, Technische Universität Berlin, Berlin, 10587 Germany; 3 School of Aeronautics and Astronautics, Zhejiang University, Hangzhou, Zhejiang 310027 China

**Keywords:** Lithium-ion battery, State of health, Ensemble learning, Model fusion, Transformer, Linear model, LSTM, Energy science and technology, Energy storage, Batteries

## Abstract

To achieve both high accuracy and interpretability in battery State-of-Health (SoH) estimation, this study proposes a dynamic time-varying multi-expert fusion network (MEFNet) framework. The framework consists of three specialized experts: a mechanism-based general expert that captures fundamental degradation patterns, an LSTM-based local expert for short-term dynamics, and a Transformer-based global expert for long-term dependencies. These experts are integrated through a novel linear dynamic weighting scheme that adapts to evolving battery health states. This fusion framework balances interpretability and accuracy while accounting for the scarcity of full lifecycle battery data, particularly addressing challenges stemming from limited real-world data collection conditions that typically only cover early-stage operations. The experimental validation demonstrates that the critical end-of-life threshold (SoH = 70% or 80%) typically occurs within the early (0-30%) to middle (30-60%) degradation stages. The proposed MEFNet achieves superior estimation accuracy using only 25% of the lifecycle data, outperforming models trained on complete datasets particularly during early and middle degradation stages.

## Introduction

Lithium-Ion batteries (LIBs) play a vital role in rapidly growing fields like renewable energy and electric vehicles, due to their high energy density, long life, and reliability^1,2^. Estimating the State of Health (SoH) of LIBs is critical for optimizing Battery Management Systems (BMS), as it impacts battery performance, lifespan, and safety^3^ and directly impacts device longevity and user experience.

**Fig. 1:**
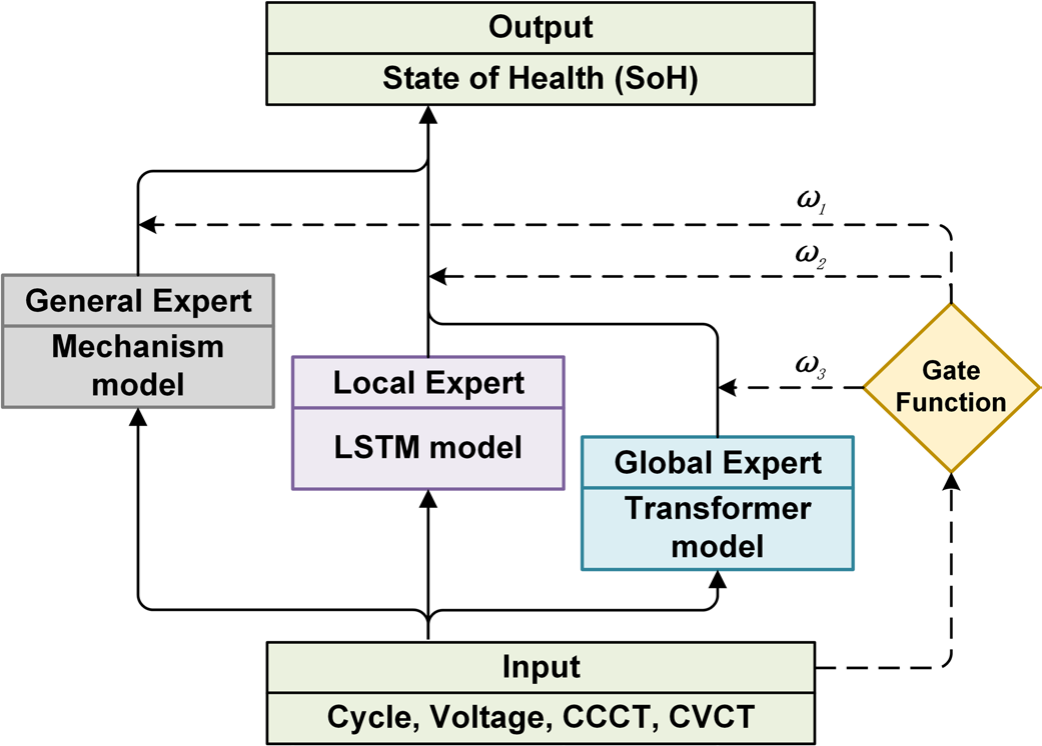
MEFNet: Multi-Expert Fusion Network.

The main current SoH of LIBs estimation methods are model-based methods and data-driven methods^4,5^. A model-based method is a mathematical model that uses physical knowledge to represent the health degradation process of LIBs and requires no data preparation. Among them, the Equivalent Circuit Models (ECMs) represent the internal electrochemical processes of batteries through components such as resistors and capacitors, offering strong physical interpretability but limited adaptability^6,7^. Electrochemical models - such as the Pseudo-Two-Dimensional (P2D) and Doyle-Fuller-Newman (DFN) models – use differential equations to depict electrochemical reactions and ion transport, achieving high accuracy at the cost of high computational complexity^8^. Degradation models, on the other hand, focus on mechanisms such as Solid-Electrolyte Interphase (SEI) formation and active material loss that lead to capacity fade; typically relying on empirical or semi-empirical methods and experimental data for long-term SoH estimations, they may lack generalizability across varying scenarios^9^. As described above, the mechanism of the LIBs is complex and not easy to be modelled. In addition, the model is flawed in terms of prediction accuracy and sensitive to external environmental interference and has poor adaptability and robustness^10^.

With advancements in computational power and increased data availability, data-driven models have become an important approach for SoH estimation^11^. Unlike model-based methods, data-driven methods require no extensive expert knowledge^12^. Instead, these methods only require the collection of aging data of LIBs, without the need to analyse the degradation mechanism of LIBs^13^. For instance, Deng et al.^14^ proposed a method combining early aging data for degradation pattern recognition and transfer learning. This model extracted features from discharge capacity for degradation pattern recognition and state of health estimation, enhancing prediction accuracy. Zhou et al.^15^ integrated a Denoising Autoencoder with a Transformer network to preprocess raw data and capture temporal information, significantly reducing prediction errors. Lin et al.^16^ decomposed LIBs’ capacity into a global declining trend and local fluctuations, employing a gated recurrent unit neural network and a hidden Markov model to capture long-term global declining trends and quantify uncertainty. Meng et al.^17^ developed a multi-scenario transferable learning framework with few-shot capability to predict LIBs lifespan trajectory. The above methods significantly improve prediction accuracy, but regarding the data-driven method, model establishment requires a large amount of data.

Moreover, data-driven methods often involve dimensionality reduction and feature engineering^18^, and may violate physical principles, becoming unrealistic and lacking interpretability. To address these interpretability concerns, there is a growing trend toward implementing physics-informed neural networks and physics-guided machine learning approaches that integrate physical principles into data-driven models^19–22^. If there is a lack of enough data for training, data-driven methods may perform poorly^23^. Therefore, data-driven methods usually require a large amount of training data to cover every situation of different batteries in order to achieve high accuracy, and training data must be prepared for each situation to cover a variety of batteries^9^. However, collecting data that accurately reflect real-world battery usage conditions poses significant challenges. While data obtained from controlled laboratory environments can be useful, they often fail to fully capture the complex operating conditions of batteries in practical applications, which are subject to temperature fluctuations, load changes, and irregular charge-discharge cycles^24^. For instance, under controlled laboratory conditions, the probability of a battery exhibiting issues during the early cycles is typically low. However, this does not guarantee that the battery will maintain an ideal SoH degradation curve during later cycle periods, especially under real-world operating conditions, such as those in industrial energy storage systems or electric (EV)^25,26^. Consequently, predicting the SoH of batteries in future cycles using data from the initial cycles becomes particularly valuable for ensuring production safety and the driving safety of EV owners. Therefore, the main question remains: How can the State of Health (SoH) estimation method be developed with limited data for enhancing battery system management efficiency?

Mechanistic models can be constructed without extensive data collection and offer valuable interpretability, but often perform poorly under complex real-world conditions. In contrast, data-driven methods offer greater flexibility and accuracy, but are often limited by data availability and quality. In addition, Long Short-Term Memory (LSTM) cyclically retains information from previous time steps through memory units, enabling learning of data context, but they still struggle to capture long-range dependencies when dealing with particularly long sequences^27^. Transformer on the other hand relies on attention mechanisms that allow direct information exchange between any two positions in the sequence, making it more suitable for handling long-range dependencies^28^. To address these challenges and limitations, we propose Multi-Expert Fusion Network (MEFNet) as shown in Fig. 1, a dynamic time-varying hybrid multi-expert architecture based on an ensemble learning framework. Unlike direct capacity calculations that provide instantaneous SoH, our approach enables predictive modeling of future battery health trajectories using limited early-stage data, facilitating proactive maintenance strategies. MEFNet includes a mechanisms expert, an LSTM expert and a transformer expert. By combining the strengths of different experts, MEFNet ensures stable health state estimation, especially in scenarios with limited early stage data for lithium-ion batteries. MEFNet bridges the gap between mechanistic models and data-driven methods, providing a more robust and adaptable solution for state of health estimation. The main contributions can be summarized as follows:

- A framework based on ensemble learning is proposed for predicting the SoH of LIBs, where the mechanism model serves as a general expert, while LSTM and Transformer in the data-driven method function as local and global experts, respectively.
- A dynamic gating function for expert weight allocation is developed, which varies over time according to input data characteristics and battery health. Finally, the outputs of different experts are fused to optimize the overall prediction accuracy.
- Quantitative experiments demonstrate that our method can achieve high accuracy even when there is limited data. For example, in the CALCE and NASA datasets, the best results can be achieved using only 25% of the available data.

The remainder of this paper is structured as follows. Section "Data analysis and processing" introduces the dataset utilized in this study, along with the data processing techniques applied to ensure data integrity and consistency. Section “Proposed method” details the proposed Multi-Expert Fusion Network (MEFNet) framework, including the mechanism-based general expert, the LSTM-based local expert, the Transformer-based global expert, and the dynamic weighting strategy for fusion. Section "Experiment result and discussion" presents the experimental setup, covering baseline approaches, evaluation metrics, and dataset partitioning, followed by an in-depth analysis of the results. Additionally, ablation studies are conducted to assess the contributions of individual components. Finally, Sect. "Discussion and conclusion" concludes the study concludes with a discussion on key findings and potential directions for future research.

## Data analysis and processing

### Dataset introduction

In our study, we performed experiments on two publicly available datasets CALCE and NASA. CALCE from the Center for Advanced Life Cycle Engineering (CALCE) at the University of Maryland^29–31^ and NASA from the NASA Ames Research Center^32,33^, both offering valuable insights into lithium-ion battery behavior and performance. Each battery includes data from undergoing repeated cycles of charging, discharging, and impedance measurement under controlled conditions. To ensure experimental rigor and consistency, we specifically selected $\text {LiCo}_2$ battery data from these datasets under controlled CCCV charging conditions. This deliberate design choice eliminates confounding variables from different battery chemistries and test protocols, establishing a rigorous baseline for evaluating the multi-expert fusion approach. The controlled conditions ensure that the model learns actual degradation patterns rather than test condition variations, while the limited yet comprehensive nature of the selected data enables focused and detailed analysis.

### Data processing

The key features used in this study, including Discharge Voltage (DV), Constant Voltage Charging Time (CVCT), Constant Current Charging Time (CCCT), and Cycle Count (${N}_\text {c}$), are obtained by simple processing of the raw data in the dataset. These features are recorded under controlled experimental conditions and serve as critical indicators of the battery’s performance and aging state.

- **DV** reflects the battery voltage measured during discharge. It indicates the battery’s ability to sustain voltage under load and is influenced by internal resistance and capacity degradation.
- **CVCT** measures the duration of the constant voltage phase during charging, which decreases with capacity degradation.
- **CCCT** represents the duration of the constant current phase during charging, reflecting the charging efficiency and state of health.
- **Cycle Count** provides a cumulative record of charge-discharge cycles, directly correlating with battery aging.

In data preparation, we obtained the voltage for each cycle during the discharge period from the raw data and calculated its average value to determine the DV for each cycle; obtain the CCCT and CVCT from the current-voltage curve in the raw data; and calculate the SoH of the battery for each cycle based on the rated capacity ${C_{\text {rated}}}$ and current capacity ${C_{\text {current}}}$ of the battery in the raw data, which is used for loss calculation in training and result evaluation of the final test set.

1 $$\begin{aligned} SoH = \frac{C_{\text {current}}}{C_{\text {rated}}} \times 100\% \end{aligned}$$

where ${C_{\text {rated}}}$ is the manufacturer’s nominal capacity and ${C_{\text {current}}}$ is the cycle-wise discharge capacity described above. To ensure data integrity for subsequent experiments, we eliminated outliers by identifying values deviating from the mean by more than one standard deviation using a sliding window, filtering sudden changes exceeding a threshold, and resetting the index. Next, we normalized all features to the range [0,1] using MinMax scaling fitted only on the training set, then applied the same scaler to validation and test sets. Missing values were handled through linear interpolation.

## Proposed method

### Framerwork

**Fig. 2:**
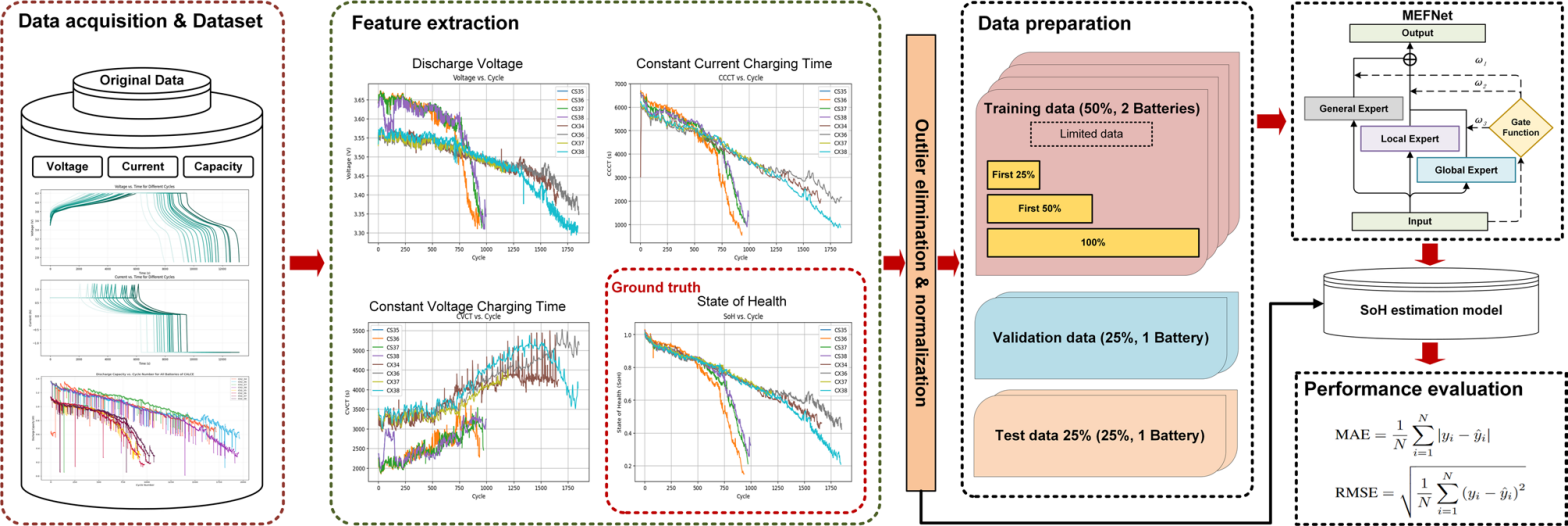
Flowchart of the MEFNet-based SoH estimation process for LIBs.

The proposed method for SoH estimation follows a structured pipeline, as illustrated in Fig. 2. Data acquisition involves collecting multi-source data, including voltage, current, and capacity, from the LIB charging process. In feature extraction, key battery health indicators such as DV, CCCT, and CVCT are identified, with SoH derived as the ground truth for model training and validation.

The model training phase employs the proposed MEFNet, a hybrid framework designed for accurate SoH estimation. The dataset is initially split into training (50%), validation (25%), and test (25%) subsets. Within the training set (50%), further subdivisions into 25%, 50%, and 100% are implemented to analyze the impact of data availability on model performance.

Finally, performance evaluation is conducted on unseen test data, measuring accuracy using Mean Absolute Error (MAE) and Root Mean Square Error (RMSE). The extracted feature distributions further enhance model interpretability, ensuring a robust and reliable SoH estimation framework. MEFNet uses ensemble learning to combine multiple individual models to produce the final prediction result. The models of MEFNet are divided into two categories: general experts based on model methods and global and local experts based on learning methods. The results of each expert are combined and supplemented according to the characteristics of the LIBs, based on the weight distribution of the non-linear routing equation, through a weighted fusion strategy and a strategic fusion method that considers specific models to a limited extent under certain conditions, so as to fully exploit the advantages of each model in different scenarios. The detailed architecture of the model, including its hierarchical structure, expert integration mechanism, and fusion strategies, is illustrated in Fig. 2, and its key components are described in detail below.

### General expert

The General Expert Model is built on the behavior of battery mechanisms, which includes both physical and chemical behaviors. Many studies have confirmed the trend of SoH and its quadratic polynomial fitting^29,34,35^, while others have applied quadratic polynomials to predict SoH^36–38^ and attempted to explain the underlying physical and chemical mechanisms^39–41^. By leveraging knowledge of charge-discharge cycles, capacity degradation patterns, and experimental calibration data, previous research has demonstrated the effectiveness of using a quadratic polynomial framework to characterize battery aging under various operating conditions^29,34–41^. Therefore, this model also adopts a quadratic polynomial mathematical framework, providing a more intuitive physical interpretation of battery health dynamics and improved scalability across different battery chemistries and use cases.

The number of charge-discharge cycles is represented as ${N}_\text {c}$ and the SoH predicted by the General Expert is $Y_{\text {general}}$. Based on the mechanism analyzed above, the relationship between the SoH of battery and the cycle count is described by a quadratic polynomial equation:

2 $$\begin{aligned} \textbf{Y}_{\text {general}} = a \cdot {N}_\text {c}^2 + b \cdot {N}_\text {c} + c \end{aligned}$$

where $a$, $b$, and $c$ are coefficients determined through experimental fitting. According to sensitive review studies to the End of Life (EoL) of battery criteria considered^42^, manufacturers generally recommend replacing batteries when their SoH drops to 70–80% of the nominal capacity, as the available capacity is expected to decrease at a faster rate after EoL^43^.

To calculate these coefficients of equation (2) to determine its mechanism model, we constructed an equation based on data from the battery in three key states, namely SoH of 100%, 70% and 80%. These three points form a linear system that can be solved to determine the coefficients a, b, and c through standard linear algebra methods. This experimental fitting approach ensures the quadratic model is calibrated using actual measured degradation data for each specific battery type.

The coefficients are determined separately for each battery model used in our work. The General Expert Model combines theoretical rigor with practical calibration, offering significant advantages over purely data-driven methods. In practical deployments, a simple second-order mechanism model can be constructed using LIBs manufacturer laboratory data. By incorporating the behavioral mechanism underlying battery aging, it enhances interpretability, scalability, and adaptability across varying operational scenarios.

**Fig. 3:**
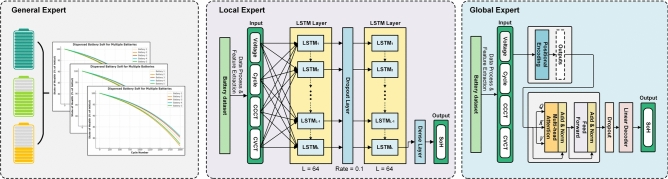
Detailed structure of the MEFNet model, which is based on a mechanism model for general experts, LSTM-based local experts and Transformer-based global experts.

### Local expert

The LSTM-based local expert is a data driving module, responsible for processing multidimensional local battery features and predicting the corresponding SoH^27^. As illustrated in Fig. 3, the architecture comprises the following components: The first LSTM layer (64 units) preserves the full time-sequence information and incorporates a 10% dropout rate to reduce overfitting. The second LSTM layer (64 units) focuses on extracting global dependencies by returning only the final hidden state. Finally, a fully connected layer maps this hidden state to the SoH prediction. The input feature sequence is defined as:

3 $$\begin{aligned} \textbf{X} = \{ \text {X}_{\text {DV}}, \text {X}_{\text {CCCT}}, \text {X}_{\text {CVCT}}, \text {X}_{\text {Cycle}} \} \end{aligned}$$

where each $\textbf{X}_t$ at time step $t$ includes battery state features. After data normalisation and reshaping, the input sequence is fed into a two-layer LSTM network.

Finally, this final hidden state is fed into a fully connected layer to obtain the local SoH estimate, thus completing the local expert’s prediction of LIBs SoH.

### Global expert

The global expert architecture depicted in the Fig. 3 employs a Transformer-based model to predict the State of Health (SoH) by capturing global patterns and long-range dependencies in time series data, its multi-head attention mechanism can better capture the complex nonlinear characteristics of the sequence^28^. As another data-driven model, the global expert receives the same input specified in equation (3) as the local expert. However, since Transformers inherently lack positional information, positional encoding (PE) is introduced to enhance the input features and enable the model to differentiate sequence positions:

4 $$\begin{aligned} \textbf{X}' = \textbf{X} + \text {PE}(\textbf{X}) \end{aligned}$$

where $\textbf{PE}$ denotes the positional encoding. This allows the model to identify the relative position of each feature within the time series. The position-encoded $\textbf{X}'$ is then used to compute the query $\mathbf {(Q)}$, key $\mathbf {(K)}$, and value $\mathbf {(V)}$ matrices as follows:

5 $$\begin{aligned} \textbf{Q} = \textbf{X}' \textbf{W}^Q, \quad \textbf{K} = \textbf{X}' \textbf{W}^K, \quad \textbf{V} = \textbf{X}' \textbf{W}^V \end{aligned}$$

where $\textbf{W}^Q$, $\textbf{W}^K$, and $\textbf{W}^V$ are learnable weight matrices for linear transformations. These matrices represent the query, key, and value for the attention mechanism. The relevance between time steps is computed by applying the scaled dot-product attention mechanism, where the score matrix is scaled by a factor of $\sqrt{d_k}$ to control the range of the scores and stabilize training. The softmax operation is then used to obtain the attention weight matrix and generate the time step features:

6 $$\begin{aligned} \textbf{Z} = \text {Attention}(\textbf{Q}, \textbf{K}, \textbf{V}) = \text {softmax}\left( \frac{\textbf{Q} \textbf{K}^\top }{\sqrt{d_k}}\right) \textbf{V} \end{aligned}$$

The representation $\textbf{Z}$ is then calculated as the result of equation (6). To preserve the original feature information and stabilize training, a residual connection is applied between $\textbf{Z}$ and the input $\textbf{X}'$. The result is normalized to produce $\textbf{Z}'$, which is further processed through a feedforward network (FFN) consisting of two fully connected layers for non-linear feature extraction and feature representation:

7 $$\begin{aligned} \textbf{Z}' = \text {LayerNorm}(\textbf{Z} + \textbf{X}'), \quad \textbf{F}_{\text {global}} = \text {FFN}(\textbf{Z}') \end{aligned}$$

The global feature representation $\textbf{F}_{\text {global}}$ from all time steps is then pooled to aggregate the sequence’s features into a single global feature vector, $\textbf{f}_{\text {global}}$:

8 $$\begin{aligned} \textbf{f}_{\text {global}} = \frac{1}{T} \sum _{t=1}^{T} \textbf{F}_{\text {global}}[t] \end{aligned}$$

Finally, the global feature vector $\textbf{f}_{\text {global}}$ is passed through a linear layer to predict the SoH, denoted as $\textbf{Y}_{\text {global}}$:

9 $$\begin{aligned} \textbf{Y}_{\text {global}} = \textbf{W}_{\text {p}} \textbf{f}_{\text {global}} + \textbf{b}_{\text {p}} \end{aligned}$$

where $\textbf{W}_{\text {p}}$ and $\textbf{b}_{\text {p}}$ are the learnable weight matrix and bias of the output layer.

### Gate function

The Gate Function is a dynamic weight allocation mechanism that adaptively combines the contributions of the general expert (mechanism-based model), local expert (LSTM), and global expert (Transformer). It employs a nonlinear function that evolves with the number of iterations to allocate weights dynamically. The final SoH prediction result of MEFNet is obtained as a weighted sum of the three expert models:

10 $$\begin{aligned} \textbf{Y} = w_1(N_\text {c}) \cdot \textbf{Y}_{\text {general}} + w_2(N_\text {c}) \cdot \textbf{Y}_{\text {local}} + w_3(N_\text {c}) \cdot \textbf{Y}_{\text {global}} \end{aligned}$$

where $\textbf{Y}$ is the predicted SoH of the lithium-ion battery (LIB), and the weights satisfy the following constraints:

11 $$\begin{aligned} & w_1(N_\text {c}) + w_2(N_\text {c}) + w_3(N_\text {c}) = 1 \end{aligned}$$

12 $$\begin{aligned} & w_1(N_\text {c}), w_2(N_\text {c}), w_3(N_\text {c}) \ge 0 \end{aligned}$$

Here, $w_i(N_\text {c})$ represents the dynamic weight for each expert. In analyzing the SoH degradation patterns of LIBs, the early stage is characterized by a relatively stable and approximately linear decay process. However, as time or cycle count increases, the degradation enters an accelerated phase marked by pronounced nonlinear behavior. This accelerated decay is primarily caused by the irreversible accumulation of chemical and physical damage within the battery. Once this accumulation surpasses a certain threshold, the degradation rate increases significantly^44,45^. To capture these nonlinear acceleration features, a quadratic function is employed. Additionally, to ensure smoother transitions and avoid abrupt changes between different experts’ outputs, we adopt the $(N_{\text {c}}/2T_{\text {c}})^2$, which is both simple and effective in reflecting accelerated nonlinearity.

Transformer^28,46,47^, as a data-driven model known for its outstanding performance in image and sequence data prediction, excels at capturing complex data features. Thus, in this work, the global expert (Transformer) is designated as the primary model, receiving the majority weight. Meanwhile, LSTM demonstrates strong performance in short-term predictions but tends to accumulate errors in long-term forecasting^27,48,49^. On the other hand, the mechanism-based model, while effective at capturing general trends, becomes increasingly valuable as the battery ages and exhibits more pronounced degradation patterns that align with theoretical expectations.On the other hand, the mechanism-based model, while effective at capturing general trends, becomes increasingly valuable as the battery ages and exhibits more pronounced degradation patterns that align with theoretical expectations.

Based on extensive empirical testing and domain expertise in battery degradation behavior, we developed a weight distribution function that balances the contributions of each expert according to the progression of battery aging. This function increases the relative importance of the mechanism-based and local models as the cycle count increases, while allowing the global expert to dominate during early cycles when degradation behavior is more regular and predictable:

13 $$\begin{aligned} {\left\{ \begin{array}{ll} w_1(N_{\text {c}}) = 0.5 \cdot \left( {N_{\text {c}}}/{2T_{\text {c}}}\right) ^2 \\ w_2(N_{\text {c}}) = 1 - \left( {N_{\text {c}}}/{2T_{\text {c}}}\right) ^2 \\ w_3(N_{\text {c}}) = 0.5 \cdot \left( {N_{\text {c}}}/{2T_{\text {c}}}\right) ^2 \end{array}\right. } \end{aligned}$$

where ${N_{\text {c}}}$ is current number of cycle and ${T_{\text {c}}}$ is the total number of cycles that the battery needs to predict. This formulation ensures that the global expert (Transformer) receives the highest weight (${w_2}$) during the initial cycles, with its contribution gradually decreasing as ${N_{\text {c}}}$ approaches ${T_{\text {c}}}$. Concurrently, the general and local experts gain increasing importance as the battery ages, with each receiving a maximum weight of 0.125 at ${N_{\text {c}}} = {T_{\text {c}}}$. For experimental validation, the ${T_{\text {c}}}$ of known batteries equals the actual count from the dataset. This allows validation of MEFNet’s full lifecycle prediction capability. For practical deployment of new batteries, Tc is calculated using the general expert by solving ${SoH(N) = SoH_{thr}}$, where ${SoH_{thr}}$ is the end-of-life threshold (typically 70%−80%).

14 $$\begin{aligned} T_c=\frac{-b+\sqrt{b^2-4a(c-SoH_{thr})}}{2a} \end{aligned}$$

This adaptive weighting strategy was determined through systematic experimental validation and reflects the changing reliability of each expert’s predictions throughout the battery lifecycle. Our testing confirmed that this gating function effectively integrates the strengths of each expert.

### Learning of data-driven expert

We use mean squared error (MSE) as the loss function for the local expert (LSTM) and global expert (Transformer). The loss function $\mathcal {L}(\theta )$ is defined as:

15 $$\begin{aligned} \mathcal {L}(\theta ) \;=\; \frac{1}{N}\,\sum _{i=1}^{N}\bigl (\hat{s}_{i} - s_{i}\bigr )^2 \end{aligned}$$

where $\theta$ represents the set of parameters of the model, $\hat{s}_{i}$ and $s_{i}$ denote the predicted and actual values, respectively.

**Fig. 4:**
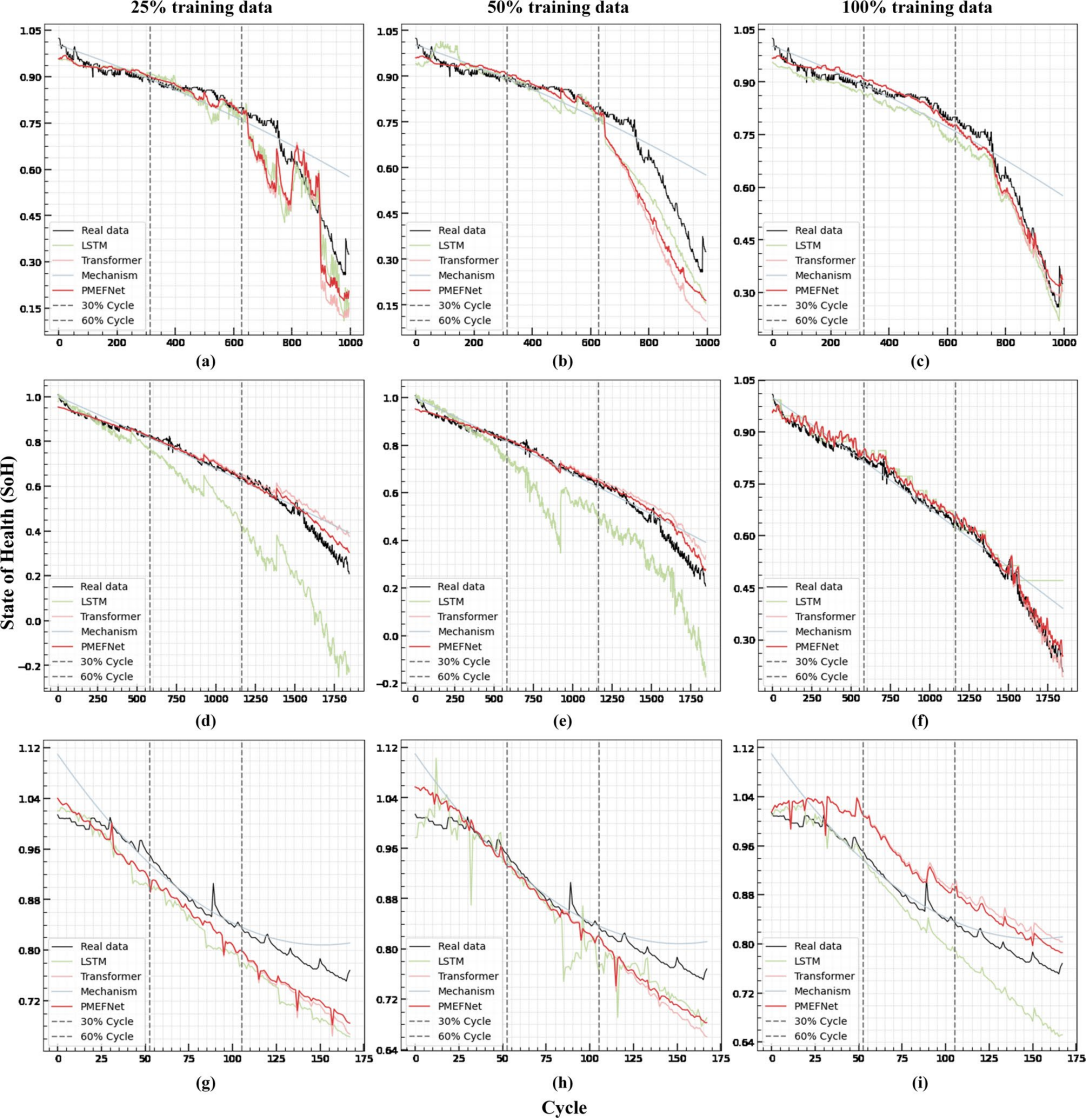
Comparison of different models’ trajectories of SoH estimation of CX 1350 mA, CS 1100 mA and B 1865 mA on 25%, 50% and 100% of data set for training by different models. (a) (b) (c) CS 1100 mA by different methods on 25%, 50% and 100% of data. (d) (e) (f) CX 1350 mA by different methods on 25%, 50% and 100% data. (g) (h) (i) B 1865 mA by different methods on 25%, 50% and 100% data.

## Experiment result and discussion

To ensure data consistency, the training, validation, and test data for this experiment all come from batteries of the same model. Additionally, training with limited data simulates real-world scenarios with insufficient data, exploring the model’s adaptability and predictive capability under constrained conditions. The experiment primarily analyzes the model’s efficiency in utilizing early battery data and evaluates its ability to predict full lifecycle performance from short-cycle data. Furthermore, the performance differences of models trained with varying cycle lengths on validation and test sets further assess their generalization ability. In this experiment, batteries of the same model are selected from two datasets, with each scenario including four batteries: two for training, one for validation, and one for testing. Our experimental design begins with the limitation of the training data in the original dataset. To demonstrate the generality of our model, we arranged our data in chronological order instead of randomly sampling it from the entire dataset. The first 25%, 50%, and 100% of the data come from a single battery’s life cycle, respectively. This prevents information leakage across temporal boundaries. The detailed distribution is shown in Table 1.

**Table 1:** Data segmentation on three batteries(number of cycles).

Dataset	Ratio	Training	Validation	Test
CALCE: CS 1350 mA	25%	484	243	997
	50%	967	487	997
	100%	1934	973	997
CALCE: CX 1100 mA	25%	880	301	1845
	50%	1760	601	1845
	100%	3519	1202	1845
NASA: B 2000 mA	25%	84	42	168
	50%	168	84	168
	100%	336	168	168

Throughout the experiment, the data sets for all experiments are very small, with relatively more data in CALCE and relatively fewer cycles in the NASA data set. Our proposed model will be validated in CALCE and then in the NASA data for higher-level performance validation.

**Table 2:** The results for different training data volumes in the CALCE dataset are compared according to different cycle stages.

Dataset	Percentage of data used for training	Different stages of cycle	Mechanism		LSTM		Transformer		MEFNet	
			MAE	RMSE	MAE	RMSE	MAE	RMSE	MAE	RMSE
CALCE CX 1350mA	25%	0% – 30%	0.143	0.024	0.151	0.027	0.098	0.014	**0.098**	**0.014**
		30% – 60%	0.108	0.014	0.335	0.118	0.111	0.016	**0.102**	**0.014**
		0% – 60%	0.127	0.020	0.260	0.085	0.104	0.015	**0.100**	**0.014**
		60% – 100%	0.227	0.071	0.557	0.322	0.248	0.079	**0.187**	**0.043**
		0% – 100%	0.174	0.047	0.406	0.214	0.176	0.051	**0.141**	**0.029**
	50%	0% – 30%	0.143	0.024	0.165	0.032	**0.107**	**0.015**	**0.107**	**0.015**
		30% – 60%	0.108	0.014	0.381	0.159	0.099	0.013	**0.094**	**0.011**
		0% – 60%	0.127	0.020	0.294	0.115	0.103	0.014	**0.101**	**0.014**
		60% – 100%	0.227	0.071	0.431	0.197	0.248	0.074	**0.198**	**0.050**
		0% – 100%	0.174	0.047	0.355	0.153	0.176	0.048	**0.147**	**0.033**
	100%	0% – 30%	0.143	0.024	**0.139**	**0.023**	0.148	0.025	0.147	0.025
		30% – 60%	**0.108**	**0.014**	0.161	0.029	0.139	0.021	0.136	0.021
		0% – 60%	**0.127**	**0.020**	0.151	0.026	0.143	0.023	0.142	0.023
		60% – 100%	0.227	0.071	0.258	0.096	**0.131**	**0.021**	0.141	**0.021**
		0% – 100%	0.174	0.047	0.201	0.064	**0.138**	**0.023**	0.142	0.024
CALCE CS 1100mA	25%	0% – 30%	0.139	0.023	0.131	0.022	0.127	0.020	**0.127**	**0.020**
		30% – 60%	0.151	0.028	0.171	0.038	**0.125**	**0.020**	**0.125**	**0.020**
		0% – 60%	0.145	0.026	0.152	0.031	**0.126**	**0.020**	**0.126**	**0.020**
		60% – 100%	0.324	0.141	0.296	0.106	0.334	0.133	**0.307**	**0.111**
		0% – 100%	0.234	0.092	0.221	**0.071**	0.233	0.086	**0.218**	**0.071**
	50%	0% – 30%	0.139	0.023	0.174	0.038	**0.136**	**0.022**	**0.136**	**0.022**
		30% – 60%	0.234	0.028	0.167	0.037	**0.116**	**0.016**	**0.116**	**0.016**
		0% – 60%	0.145	0.026	0.170	0.037	**0.126**	**0.019**	**0.126**	**0.019**
		60% – 100%	0.324	0.141	**0.320**	**0.109**	0.403	0.178	0.365	0.145
		0% – 100%	**0.234**	0.092	0.241	**0.075**	0.273	0.113	0.251	0.093
	100%	0% – 30%	0.139	0.023	0.153	0.027	0.136	**0.022**	**0.135**	**0.022**
		30% – 60%	0.151	0.028	0.195	0.040	**0.115**	**0.016**	**0.115**	**0.016**
		0% – 60%	0.145	0.026	0.175	0.034	0.126	0.019	**0.126**	**0.019**
		60% – 100%	0.324	0.141	0.227	0.054	0.177	0.036	**0.170**	**0.033**
		0% – 100%	0.234	0.092	0.198	0.043	0.149	0.027	**0.146**	**0.025**

**Table 3:** The results for different training data volumes in the NASA dataset are compared according to different cycle stages.

Dataset	Percentage of data **used for training**	Different stages **of cycle**	** Mechanism**		** LSTM**		** Transformer**		** MEFNet**	
			MAE	RMSE	MAE	RMSE	MAE	RMSE	MAE	RMSE
NASA B - 2000mA	25%	0% – 30%	0.186	0.047	0.159	0.030	**0.150**	**0.025**	**0.150**	**0.025**
		30% – 60%	**0.082**	**0.010**	0.181	0.037	0.164	0.030	0.163	0.029
		0% – 60%	**0.144**	0.034	0.181	0.037	0.157	0.027	0.157	**0.027**
		60% – 100%	**0.153**	**0.029**	0.201	0.043	0.242	0.060	0.228	0.053
		0% – 100%	**0.148**	**0.032**	0.267	0.073	0.196	0.044	0.189	0.040
	50%	0% – 30%	0.186	0.047	**0.138**	0.030	0.151	**0.029**	0.151	**0.029**
		30% – 60%	**0.082**	**0.010**	0.170	0.044	0.110	0.015	0.109	0.015
		0% – 60%	0.144	0.034	0.155	0.038	**0.132**	**0.023**	**0.132**	**0.023**
		60% – 100%	**0.153**	**0.029**	0.215	0.049	0.238	0.061	0.219	0.051
		0% – 100%	**0.148**	**0.032**	0.182	0.043	0.182	0.043	0.173	0.037
	100%	0% – 30%	0.186	0.047	**0.104**	**0.015**	0.186	0.038	0.186	0.037
		30% – 60%	0.082	0.010	**0.153**	**0.027**	0.244	0.060	0.236	0.057
		0% – 60%	0.144	0.034	**0.131**	**0.022**	0.217	0.050	0.213	0.048
		60% – 100%	0.153	0.029	**0.270**	**0.076**	0.232	0.054	0.203	0.042
		0% – 100%	**0.148**	**0.032**	0.199	0.051	0.223	0.052	0.209	0.046

### Baseline approaches and parameter settings

To verify that effectiveness of our proposed model can estimation SoH with limited data, we compared our model with the following baseline approaches:

- Mechanism model: The mechanism model is general expert of MEFNet and predicts battery State of Health (SoH) using a quadratic equation $SoH = a \cdot C^{2}+b\cdot C+c$. This model leverages the battery life curve provided by manufacturers, effectively capturing SoH trends during early and mid lifecycle stages. For specific construction details refer to section 3.2.
- LSTM: LSTM is also a local expert in MEFNet, which incorporates memory cells and gates (forget, input, and output) to retain and selectively update information from previous time steps, is designed to learn dependencies and temporal trends in time-series data. This model is particularly suited for capturing local features in battery cycle data. LSTM has two key hyperparameters: learning rate and the number of hidden layers. The learning rate is set at 0.0003, and the number of hidden layers is set at 2, with each layer containing 64 units.
- Transformer: Transformer, which uses a self-attention mechanism, captures global dependencies across entire input sequences and is global expert of MEFNet. Key hyperparameters include the learning rate and number of attention heads, with a learning rate of 0.0003 and 2 attention heads used in this study.

**Table 4:** Ablation studies of the performance of the proposed MEFNet components in different cycle stages using the CALCA dataset.

Different stages of cycle	General Expert		Local Expert		Global Expert		Local + General Expert		Global + General Expert		MEFNet	
	MAE	RMSE	MAE	RMSE	MAE	RMSE	MAE	RMSE	MAE	RMSE	MAE	RMSE
0% – 30%	0.143	0.024	0.151	0.027	**0.098**	**0.014**	0.150	0.027	**0.098**	**0.014**	**0.098**	**0.014**
30% – 60%	0.108	**0.014**	0.335	0.118	0.111	0.016	0.327	0.111	0.107	0.015	**0.102**	**0.014**
0% – 60%	0.127	0.020	0.260	0.085	0.104	0.015	0.254	0.081	0.103	**0.014**	**0.100**	**0.014**
60% – 100%	0.227	0.071	0.557	0.322	0.248	0.079	0.498	0.253	0.243	0.077	**0.107**	**0.043**
0% – 100%	0.174	0.047	0.406	0.214	0.176	0.051	0.371	0.172	0.173	0.050	**0.141**	**0.029**

### Evaluation metrics

We evaluate the performance of our model using MAE and RMSE, which are mathematically defined as follows:

16 $$\begin{aligned} \text {MAE}= & \frac{1}{N} \sum _{i=1}^{N} \left| y_i - \hat{y}_i \right| \end{aligned}$$

17 $$\begin{aligned} \text {RMSE}= & \sqrt{\frac{1}{N} \sum _{i=1}^{N} \left( y_i - \hat{y}_i \right) ^2} \end{aligned}$$

Where $y_i$ is the true value; $\hat{y}_i$ is the predicted value; $N$ is the sample size. These metrics quantify the model’s predictive performance on the normalized SoH scale (0–1). MAE reflects the average magnitude of errors, while RMSE is more sensitive to larger errors.

### Result analysis

From the results in Table 2, it can be seen that if only 25% of the data is used for training, MEFNet achieves the lowest MAE and RMSE both for segmented cycle ranges and for the entire cycle on the CX and CS batteries of CALCE. In the CX battery, MEFNet with 25% of the training data can reach the level of MEFNet and Transformer with nearly 100% of the training data. Moreover, when the training ratio increases to 50%, the model’s performance remains relatively stable at or near the minimum error level, indicating MEFNet’s strong generalization capability under limited data conditions.

When training data are increased to 100%, the performance of MEFNet in terms of MAE and RMSE becomes somewhat less competitive for the CX battery compared to other methods; however, it remains superior for the CS battery. This phenomenon, while appearing counterintuitive, can be attributed to the fundamental design philosophy of MEFNet. The model was specifically engineered to excel in limited data scenarios by leveraging the complementary strengths of its three expert components. With 100% training data, the data-driven components (especially the Transformer) may overfit to specific patterns in the training data that don’t generalize well to test conditions, particularly for batteries like CX that exhibit more consistent degradation patterns. In contrast, when training with less data (25% or 50%), MEFNet maintains a more balanced integration of mechanism-based knowledge and data-driven insights, resulting in more robust predictions.

As shown in Fig. 4 (c) and (f), the SoH-trajectory of the CS battery exhibits a more rapid and irregular decline in the later stages (60%–100%). MEFNet effectively captures such particular data characteristics and accurately tracks the degradation trend. This highlights one of MEFNet’s key advantages: its ability to adapt to irregular degradation patterns through its dynamic gate function, which gradually increases the contribution of mechanism-based and local expertise as cycles progress. Compared with other methods, it more smoothly follows the actual curve, achieves lower error, and is thus more suitable for real-world applications where battery degradation patterns are often unpredictable and irregular.

According to the results shown in Table 2 and Fig. 4 (a) - (f), the overall performance of MEFNet is outstanding in terms of both small samples and different life segments. It not only reflects its low dependence on training data, which is a significant advantage in practical applications where extensive historical data may be unavailable, but also shows its good prediction ability of the later decay trend when many conventional models struggle with accuracy. It is a stable and accurate battery SoH estimation model that offers consistent performance across various operating conditions and degradation patterns.

To further verify the performance and robustness of MEFNet. As shown in Table 3 and Fig. 4 (g) - (i), for Battery B of NASA dataset, MEFNet achieves relatively outstanding performance in terms of RMSE during the 0–30% cycle stage, particularly when trained on first 25% and 50% of the training data with small-sample training. In this case, its results are comparable to Transformer. However, in other cycle stages, the mechanism model demonstrates superior performance. In the case of 100% training data, the LSTM performs the best, and the results of MEFNet are relatively low compared to other models. Combined with the experimental data distribution in Table 1, this is due to the small amount of training data. Transformer is good at capturing long-term dependencies and requires a large amount of data for training. General experts are quickly obtained through experimental data and massive amounts of labeled data from the manufacturer. LSTM, as a network architecture that is simple and specializes in capturing local features, can also achieve near-optimal results even with NASA’s small amount of training data. Since MEFNet is dominated by the global expert, the above result is that MEFNet is not effective.

### Ablation studies

We will introduce ablation experiments to analyze and verify the contribution of each component of MEFNet in few shot learning. The model generated using the first 25% of the training data on the CALCE dataset was evaluated. Since the framework as a whole requires guidance from the physical mechanism, the Mechanism-based general expert is always involved in subsequent comparative experiments. The settings and results of our ablation experiments are shown in Table 4.

The final experimental results indicate that choosing Transformer based global expert is crucial. Moreover, as shown in Fig. 4, the mechanism-based general expert exhibits positive bias in certain cases, while the local expert, LSTM, demonstrates a similar trend of negative bias. According to the results in Table 4, the combination of these two experts through our gate function does not significantly improve SoH estimation. However, our developed gate function effectively integrates the capabilities of all three experts, leveraging limited early battery data to achieve accurate SoH estimation across the entire battery lifecycle. This approach overcomes the limitations of single experts or simple model combinations and demonstrates significant performance advantages.

## Discussion and conclusion

This study introduced MEFNet, a novel framework for battery State-of-Health (SoH) estimation that balances accuracy and interpretability. By integrating a mechanism-based general expert, an LSTM-based local expert, and a Transformer-based global expert through a dynamic weighting scheme, MEFNet adapts to evolving battery health conditions. Experimental results validate that MEFNet delivers satisfying SoH estimations even being trained with part of the full-life cycles data, making it feasible for real-world applications where complete degradation trajectories are often unavailable.

From the findings of this research, the integration of mechanism-based and data-driven approaches within a deep fusion framework can lead to improved prediction accuracy and model interpretability. MEFNet’s ability to surpass other models trained on incomplete datasets underscores its potential in battery diagnostics, especially for practical engineering scenarios that call for prediction with limited data. Additionally, the results confirm that ensemble learning can effectively balance domain knowledge with data-driven insights, improving robustness and adaptability. These insights provide valuable take-home messages for future researchers exploring intelligent battery health management solutions.

However, there is still room for improvement in MEFNet. The current expert models can be further optimized by incorporating more efficient and lightweight alternatives to enhance computational efficiency. As an integrated framework, MEFNet involves higher computational overhead compared to single-model approaches, requiring comprehensive computational cost analysis for practical deployment guidance. Moreover, the gate function used for fusion could be refined into a more intelligent, adaptive mechanism that dynamically adjusts weights based on specific data conditions. Future research will focus on: (1) developing adaptive gate functions, (2) exploring advanced lightweight models and model distillation techniques to reduce computational requirements, and (3) conducting comprehensive computational cost analysis to guide practical deployment in resource-constrained battery management systems.

## Data Availability

The datasets used and/or analyzed in this study are available from the authors upon reasonable request. The original datasets can be accessed via the “NASA Battery of Datasets” and “CALCE” repositories.
